# Salacinol and Related Analogs: New Leads for Type 2 Diabetes Therapeutic Candidates from the Thai Traditional Natural Medicine *Salacia chinensis*

**DOI:** 10.3390/nu7031480

**Published:** 2015-02-27

**Authors:** Toshio Morikawa, Junji Akaki, Kiyofumi Ninomiya, Eri Kinouchi, Genzoh Tanabe, Yutana Pongpiriyadacha, Masayuki Yoshikawa, Osamu Muraoka

**Affiliations:** 1 Pharmaceutical Research and Technology Institute, Kinki University, 3-4-1 Kowakae, Higashi-osaka, Osaka 577-8502, Japan; E-Mails: morikawa@kindai.ac.jp (T.M.); j.akaki@kobayashi.co.jp (J.A.); ninomiya@phar.kindai.ac.jp (K.N.); 1011610048n@kindai.ac.jp (E.K.); m-yoshikawa@leto.eonet.ne.jp (M.Y.); 2 Antiaging Center, Kinki University, 3-4-1 Kowakae, Higashi-osaka, Osaka 577-8502, Japan; 3 Faculty of Pharmacy, Kinki University, 3-4-1 Kowakae, Higashi-osaka, Osaka 577-8502, Japan; E-Mail: g-tanabe@phar.kindai.ac.jp; 4 Central R & D Laboratory, Kobayashi Pharmaceutical Co., Ltd., 1-30-3, Toyokawa, Ibaraki, Osaka 567-0057, Japan; 5 Faculty of Science and Technology, Rajamangala University of Technology Srivijaya, Thungyai, Nakhon Si Thammarat 80240, Thailand; E-Mail: yutanap@hotmail.com

**Keywords:** type 2 diabetes therapeutic candidate, *Salacia chinensis*, α-glucosidase inhibitor

## Abstract

The antidiabetic effect of a hot water extract of stems of *Salacia chinensis* (SCE) was evaluated *in vivo* in KK-A^y^ mice, a typical type 2 diabetes mellitus mice model. Administration of CE-2 dietary feed containing 0.25 and/or 0.50% of SCE for three weeks to KK-A^y^ mice significantly suppressed the elevation of both blood glucose and HbA1c levels without significant changes in body weight or food intake. Glucose tolerance was improved by administration to KK-A^y^ mice for 27 days of AIN93M purified dietary feed containing 0.12% of SCE. No suppressive effect with respect to HbA1c level was observed when AIN93M/Glc dietary feed in which all digestible glucides were replaced with glucose was administered with SCE. Thus, α-glucosidase inhibitory activity approved as the mechanism of action of the antidiabetic effect of SCE by *in vitro* investigation was reconfirmed also in *in vivo* studies. Evaluation of the α-glucosidase inhibitory activity of the active constituents, salacinol (**1**), kotalanol (**3**), and neokotalanol (**4**), by employing human α-glucosidases revealed that these compounds inhibited them as potently (IC_50_ = 3.9–4.9 μM for maltase) as they inhibited rat small intestinal α-glucosidase. The principal sulfonium constituents (**1**–**4**) were highly stable in an artificial gastric juice. In addition, **1**–**4** were hardly absorbed from the intestine in an experiment using the *in situ* rat ligated intestinal loop model. The results indicate that these sulfoniums are promising leads for a new type of anti-diabetic agents.

## 1. Introduction

**Figure 1:**
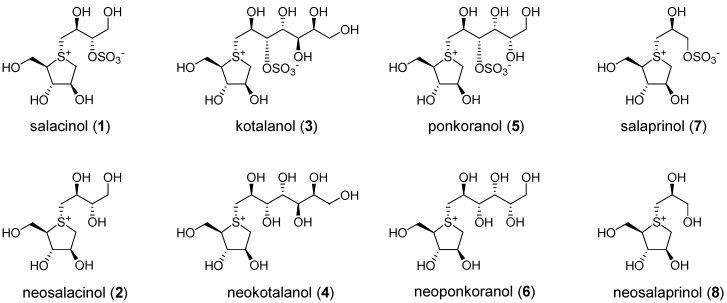
Sulfonium constituents (**1**–**8**) from the genus *Salacia* plants.

A healthful eating pattern, adequate nutrients, regular physical activity, and often pharmacotherapy are key components of diabetes management [1]. Based on a large number of chemical and pharmacological research work, numerous bioactive compounds have been found in nutritious herbal food ingredients for diabetes [2]. The genus *Salacia* (*Hippocrateaceae*) contains woody climbing plants and is widely distributed in countries, such as India, Sri Lanka, China, and Thailand. The stems and roots of the plants have been used for the prevention or cure of diabetes in these countries [3,4,5,6,7]. From a methanol and/or 80% aqueous methanol extract of the genus *Salacia* plants, we isolated a novel thiosugar sulfonium sulfate inner salt, salacinol (**1**) [7,8], as a potent α-glucosidase inhibitor. The inhibitory activities of **1** against rat small intestinal maltase and sucrose were as potent as those of acarbose or voglibose, widely used clinical inhibitors. We also isolated its analogs, neosalacinol (**2**) [9,10], kotalanol (**3**) [11,12], neokotalanol (**4**) [13], ponkoranol (**5**) [14], neoponkoranol (**6**) [15], salaprinol (**7**) [14,16], and neosalaprinol (**8**) [15], and found that compounds **2**–**6** were as potent as **1** (Figure 1). Methanol, 80% aqueous methanol, and aqueous extracts from the roots and stems of *S. reticulata* had been shown to suppress the increase in blood glucose levels in maltose, sucrose, and starch-loaded rats [6,7,17,18,19,20]. An 80% aqueous methanol extract of the roots of *S. oblonga* and stems of *S. chinensis* also exhibited anti-hyperglycemic activity in maltose and sucrose-loaded rats [18]. Several clinical trials of the *Salacia* extracts have demonstrated the efficacy of *S. retuculata* [21,22] and *S. oblonga* [23] to the patient of type 2 diabetes and that of *S. chinensis* to people with high normal blood glucose and/or volunteers in borderline hyperglycemia [24]. Safety profiles of *Salacia* extracts to acute toxicity and mutagenicity [25], and those of *S. chinensis* to reproductive outcome in rats [26] have also been demonstrated. Based on these findings, interest in the genus *Salacia* plants as a possible nutraceutical product for diabetic patients is increasing, and there has been a strong demand for efficient quality control to ensure the authenticity and the active contents of these products, as well as to verify the claims on product labels. Quantitative analyses of the sulfonium constituents (**1**–**8**) in the extracts have been developed as two separate protocols using LCMS: one for the sulfonates (**1**, **3**, **5**, and **7**) [27,28] and the other for their de-*O*-sulfonates (**2**, **4**, **6**, and **8**) [28,29]. As a result, the distributions of the sulfonium constituents in stems and roots of these plants were found to differ for the different collecting areas. Compound **4** was the major constituent in samples from Thailand, whereas **1** was the major constituent in the samples from Sri Lanka and India. An effort to discriminate the species of genus *Salacia* by referring to their RNA sequence of the internal transcribed spacer (ITS) region in the nuclear ribosomal RNA gene in an authentic specimen was conducted, and a genotype characteristic of *S. chinensis*, which is distinguishable from those of *S. reticulata* and *S. oblonga* was identified [30]. In the present study, the following *in vivo* assays of hot water extracts of Thai *S. chinensis* (SCE) and/or its sulfonium constituents (**1**–**4**) were performed: (i) suppressive effects on blood glucose level elevation in starch-loaded rats; (ii) suppressive effects on elevation of both blood glucose and HbA1c levels after chronic administration to KK-A^y^ mice; (iii) improvement effect of glucose tolerance after chronic administration to KK-A^y^ mice; and (iv) stability of these sulfoniums (**1**–**4**) in the digestive organ as well as their absorption from the digestive tract.

## 2. Experimental Section

### 2.1. Materials

#### 2.1.1. Plant Material

Extracts obtained from the stem of *Salacia chinensis*, collected in southern region of Thailand, were investigated in this study. The plant material was identified by one of the authors (Yutana Pongpiriyadacha, Rajamangala University of Technology Srivijaya). A voucher specimen of this plant is on file in our laboratory.

Hot water extraction of the de-*O*-sulfonated constituents (**2**, **4**, **6**, and **8**) was more efficient than those by methanol and aqueous methanol [28,29]. Dried stems of *S. chinensis* were crushed and extracted with hot water. The aqueous extract was evaporated under reduced pressure to obtain a hot water extract powder (abbreviated as SCE), which was used with no additives.

#### 2.1.2. Animals

Male Sprague-Dawley (SD) rats were purchased from Japan SLC, Inc., Shizuoka, Japan, and male KK-A^y^ mice were from CLEA Japan, Inc., Tokyo, Japan. The animals were housed at a constant temperature of 23 ± 2 °C, at 55% ± 15% humidity, and 12 h of illumination per day. All experiments were performed with conscious animals unless otherwise mentioned. The experimental protocol was approved by the Experimental Animal Research Committee at Kinki University.

### 2.2. Methods

#### 2.2.1. Effects of SCE and Sulfonium Constituents (**1**, **3**, and **4**) on Blood Glucose Levels in Starch-Loaded Rats

Five-week-old male SD rats were housed for one week in metal cages. After overnight fasting (20 h), the rats were orally administered a 5% (w/v) α-starch solution (1 g/kg) with or without a sample (SCE: 10–300 mg/kg, **1**: 0.15–1.48 mg/kg, **3**: 0.21–2.06 mg/kg, and **4**: 0.07–0.68 mg/kg) using a stomach tube. At 0, 0.5, 1.0, 2.0, and 3.0 h after the administration of α-starch, blood samples were taken from the tail vein and immediately subjected to the measurement of blood glucose using the glucose oxidase method. As a baseline, distilled water was administrated to rats as a “normal group”. Median effective dose (ED_50_) was determined by plotting the inhibition rate of incremental AUC_0–2 h_ (iAUC_0–2 h_; the AUC above baseline) *versus* corresponding inhibitor dosage.

#### 2.2.2. Effects on Blood Glucose Levels in SCE-Pretreated Starch-Loaded Rats

Five-week-old male SD rats were housed for one week in metal cages. After overnight fasting (20 h), the rats were orally administered SCE (75 mg/kg) using a stomach tube at various times (0, 0.5, 1.0, and 2.0 h) before loading of 5% (w/v) α-starch solution (1 g/kg). At 0, 0.5, 1.0, 2.0, and 3.0 h after the administration of α-starch, blood glucose levels were measured using the glucose oxidase method.

#### 2.2.3. Effects of Blood Glucose and HbA1c Levels after Three Weeks Administration of SCE in CE-2 Diet-fed KK-A^y^ Mice

Five-week-old male KK-A^y^ mice were housed for one week in individual metal cages. They were divided into four groups based on body weight, blood glucose, and HbA1c levels. The control group was fed a standard diet (CE-2, CLEA Japan, Inc., Tokyo, Japan) and the three SCE-treated groups were fed diets supplemented with 0.10, 0.25, and 0.50% (w/w) SCE, respectively. On day 15 and at the end of the treatment period, blood samples were collected from the tail vein under non-fasting conditions. Blood glucose and HbA1c levels were measured using glucose oxidase method and a DCA Vantage Analyzer^TM^ (Siemens, New York, USA), respectively. The HbA1c values were recorded as Japan Diabetes Society (JDS) values and were then converted to National Glycohemoglobin Standardization Program (NGSP) values as follows: HbA1c (NGSP) = 1.02 × HbA1c (JDS) + 0.25% [31].

#### 2.2.4. Effects of HbA1c Levels after Chronic Administration of SCE in AIN93M Purified and AIN93M/Glc (All Digestible Glucides Replaced with Glucose) Diet-fed KK-A^y^ Mice

Five-week-old male KK-A^y^ mice were housed for one week in individual metal cages. These mice were divided into two groups based on body weight, blood glucose and HbA1c levels; a control group was fed a standard diet (AIN93M purified, CLEA Japan, Inc., Tokyo, Japan) and the SCE-treated group was fed the same diet supplemented with 0.03, 0.06, or 0.12% SCE. Given that the food intake of the SCE-treated group decreased, the control group was pair-fed the amount of food consumed by the SCE-treated group. At regular intervals, blood samples were collected from the tail vein under non-fasting conditions. Blood glucose and HbA1c levels were measured using the glucose oxidase method and a DCA Vantage Analyzer^™^, respectively. In the oral glucose tolerance test, 28 days after the start of the experiment, the 0.12% SCE-treated group and the corresponding control group were orally given a D-glucose solution (2.0 g/kg) after fasting for 20 h. Blood glucose levels were measured at 0, 0.5, 1.0, 2.0, and 3.0 h after administration. To identify the α-glucosidase inhibitory activity of SCE in the chronic experiments, a customized AIN93M diet (AIN93M/Glc, Table 1), in which all the digestible glucides in AIN93M were substituted by D-glucose, was administered to the KK-A^y^ mice under the same conditions.

**Table 1:** Composition of diets.

	AIN93M Purified	AIN93M/Glc
d-Glucose	–	72.0692%
Corn starch	46.5692%	–
Dextrin	15.50%	–
Sucrose	10.00%	–
Casein	14.00%	14.00%
Powdered cellulose	5.00%	5.00%
Soybean oil	4.00%	4.00%
AIN-93 mineral mixture	3.50%	3.50%
AIN-93 vitamin mixture	1.00%	1.00%
l-Cystine	0.18%	0.18%
Choline bitartrate	0.25%	0.25%
*tert*-Butylhydroquinone	0.0008%	0.0008%
Total	100%	100%

AIN93M purified: purified diet for mature rodents by the American Institute of Nutrition committee in 1993; AIN93M/Glc: a customized AIN93M diet (all the digestible glucides in AIN93M were substituted by d-glucose).

#### 2.2.5. Effects on Human Intestinal α-Glucosidase

The experiment was performed according to the method as described in our previous report [32]. A human small intestinal microsome (batch MIC318017, purchased from BIOPREDIC International, Rennes, France) in 0.1 M maleate buffer (pH 6.0) was used to determine the activity of maltase, a small intestinal α-glucosidase. A test sample was dissolved in dimethyl sulfoxide (DMSO) and the resulting solution was diluted with 0.1 M maleate buffer to prepare the test sample solution (concentration of DMSO: 10%). A substrate solution in the maleate buffer (maltose: 74 mM, 50 μL), the test sample solution (25 μL), and the enzyme solution (25 μL) were mixed at 37 °C for 30 min and then immediately heated in boiling water for 2 min to stop the reaction. The glucose concentrations were determined using the glucose-oxidase method. The final concentration of DMSO in the test solution was 2.5% and no influence of DMSO on the inhibitory activity was detected. The intestinal α-glucosidase inhibitors (acarbose, voglibose, and miglitol) were used as reference compounds.

#### 2.2.6. Stability of Sulfonium Constituents (**1**–**4**) in Artificial Gastric Juice

A solution of SCE (1.0 mg/mL) in artificial gastric juice (components: 0.2% (w/v) NaCl and 0.32% (w/v) pepsin from porcine, pH adjusted to 1.2 with HCl) was incubated at 37 °C for 1.0 or 3.0 h. Each reaction mixture was neutralized with 1 M NaOH and then filtered by ultrafiltration using Amicon Ultra (MWCO 3000 Da, Millipore, MA, USA). The sulfonium contents (**1**–**4**) of each filtrate were measured by LCMS as previously described [27,29].

#### 2.2.7. *In Situ* Intestinal Absorption Study of Sulfonium Constituents (**1**–**4**) Using Rat Ligated Intestinal Loop Model

Five-week-old male SD rats (fasting period, 18 h) were anesthetized with thiobutabarbital (80 mg/kg, *i.p.*), and then ligated jejunal loops (*ca.* 20 cm) were prepared. The aqueous SCE solution (300 mg/2 mL/body) was injected and indwelled into the loop for 0.5 or 2.0 h. The indwelling solution was collected using saline and the sulfonium contents (**1**–**4**) were measured by LCMS as previously described [27,29]. Miglitol (1 mg/2 mL/body) and d-glucose (10 mg/2 mL/body) were used as reference compounds. The content of miglitol in corresponding indwelling solution was also measured under the following LCMS conditions: column, Inertsil ODS-3 (5 μm, 2.1 × 150 mm; GL Sciences, Tokyo, Japan); column temperature, 40 °C; mobile phase, 5 mM nonafluorovaleric acid-MeOH (95:5, v/v); flow rate, 0.2 mL/min; ionization, ESI-positive mode; SIM, *m*/*z* 208; Injection volume, 1 μL; retention time (*tR*), 7.3 min. Glucose concentration was determined by using the glucose-oxidase method.

### 2.3. Statistics

Values are expressed as means ± SEM. For statistical analysis, one-way analysis of variance followed by Student’s *t*-test or Dunnett’s test was used.

## 3. Results and Discussion

First the *in vivo* anti-hyperglycemic effects of SCE and the principal sulfonium constituents, salacinol (**1**), kotalanol (**3**), and neokotalanol (**4**) were evaluated in starch-loaded rats. As shown in Table 2, SCE significantly suppressed the increase in blood glucose levels in a dose-dependent manner (30–300 mg/kg, *p.o.*). The principal sulfoniums (**1**, **3**, and **4**) also exhibited potent activity with ED_50_ values of >2.06, 0.62, and 0.54 mg/kg, respectively.

In order to estimate the duration of the effect of SCE as the α-glucosidase inhibitor, the rats were pretreated by SCE (75 mg/kg, *p.o.*) at 0, 0.5, 1.0, and 2.0 h before administration of starch. As shown in Table 3, a group of rats, which were administrated by SCE 0.5 h before starch-loading tended to suppress the increase of blood glucose levels. On the other hand, administration of SCE 1.0 h before starch loading, no significant effect was observed. Therefore, the suppressive effect of the SCE against blood glucose elevation was estimated to last for *ca.* 0.5 h.

**Table 2:** Effects of a hot water extract of stems of *Salacia chinensis* (SCE) and principal sulfoniums (**1**, **3**, and **4**) on blood glucose levels in starch-loaded rats.

Group	Dose	Blood Glucose (mg/dL)					iAUC_0-2 h_	ED_50_
	(mg/kg)	0 h	0.5 h	1.0 h	2.0 h	3.0 h	(mg·h/dL)	(mg/kg)
Normal	—	64.6 ± 2.1	69.9 ± 2.0 ^b^	68.5 ± 3.6 ^b^	61.5 ± 5.1	62.0 ± 4.2	0.0 ± 6.0 ^b^	
Control	—	63.9 ± 3.8	126.1 ± 6.3	107.6 ± 11.4	65.9 ± 7.9	54.3 ± 5.1	59.5 ± 14.5	
SCE	10	67.8 ± 3.8	124.1 ± 6.3	106.0 ± 6.3	69.8 ± 4.9	65.6 ± 3.2	60.2 ± 9.0	94.0
	30	72.9 ± 2.8	101.9 ± 3.9 ^a^	96.8 ± 4.7	66.8 ± 2.6	61.3 ± 4.1	41.9 ± 5.7	
	100	68.0 ± 2.5	86.5 ± 7.5 ^b^	90.5 ± 4.7	68.8 ± 7.1	66.9 ± 6.0	29.3 ± 9.8	
	300	67.1 ± 2.1	81.1 ± 2.1 ^b^	79.6 ± 3.3 ^b^	63.1 ± 3.5	56.5 ± 2.9	15.4 ± 4.7 ^b^	
**1**	0.21	65.9 ± 3.7	107.3 ± 8.1	99.6 ± 6.3	69.4 ± 3.4	59.6 ± 3.4	46.3 ± 7.8	>2.06
	0.69	68.3 ± 2.1	101.5 ± 5.1 ^a^	107.4 ± 3.3	73.3 ± 4.9	56.0 ± 3.5	51.8 ± 4.3	
	2.06	69.6 ± 3.9	88.3 ± 4.7 ^b^	93.1 ± 3.0	71.4 ± 3.0	54.5 ± 4.2	33.8 ± 4.4	
**3**	0.15	67.3 ± 2.5	110.1 ± 6.1	100.0 ± 6.8	68.0 ± 4.4	61.6 ± 2.5	47.7 ± 10.2	0.62
	0.49	69.9 ± 1.9	94.9 ± 3.1 ^b^	86.5 ± 4.1	65.1 ± 4.6	62.1 ± 3.2	29.1 ± 6.1	
	1.48	69.9 ± 2.3	84.1 ± 3.6 ^b^	81.0 ± 3.0 ^b^	68.8 ± 3.9	66.3 ± 1.8	21.4 ± 5.7 ^b^	
**4**	0.07	66.8 ± 2.9	104.5 ± 6.1	108.4 ± 4.7	67.0 ± 4.9	56.0 ± 5.4	50.5 ± 7.9	0.54
	0.20	64.1 ± 2.7	99.6 ± 6.0 ^b^	102.3 ± 3.5	65.0 ± 2.6	54.6 ± 4.4	41.8 ± 4.2	
	0.68	64.8 ± 3.9	89.9 ± 5.1 ^b^	88.9 ± 6.0	63.4 ± 4.8	55.6 ± 2.4	26.3 ± 8.6 ^a^	

Values are means ± SEM (*n* = 8); Significantly different from control, ^a^ *p* < 0.05, ^b^ *p* < 0.01 (Dunnett’s test); iAUC: incremental area under the curve; ED_50_: effective dose 50.

**Table 3:** Effects of SCE on blood glucose levels in SCE-pretreated starch-loaded rats.

Group	Time of Administration	Blood Glucose (mg/dL)					
	Before Starch Loading	Before SCE Loading	After Starch Loading				
			0 h	0.5 h	1.0 h	2.0 h	3.0 h
Normal	―	―	64.4 ± 2.4	62.4 ± 3.9 ^a^	60.5 ± 3.8 ^a^	56.5 ± 2.9	57.3 ± 3.7
Control	―	―	63.8 ± 2.0	126.8 ± 4.5	93.1 ± 2.2	63.9 ± 2.8	64.0 ± 2.6
SCE (75 mg/kg)	0 h	―	63.8 ± 1.4	88.5 ± 2.4 ^a^	87.0 ± 4.6	62.6 ± 4.7	62.0 ± 3.1
	0.5 h	65.4 ± 3.7	71.1 ± 3.2	109.4 ± 8.0	92.5 ± 3.9	63.5 ± 1.9	58.3 ± 3.2
	1.0 h	65.8 ± 3.0	68.3 ± 2.1	124.3 ± 6.8	94.9 ± 6.0	65.9 ± 2.3	55.3 ± 3.5
	2.0 h	63.3 ± 2.0	63.4 ± 3.1	124.1 ± 10.6	100.3 ± 6.6	64.5 ± 3.3	63.6 ± 5.3

Values are means ± SEM (*n* = 8); Significantly different from control, ^a^ *p* < 0.01 (Dunnett’s test).

Next, effect of three weeks of administration of SCE on both blood glucose and HbA1c levels by using Kuo Kondo (KK)-yellow agouti (A^y^) mice. The KK mice originating from Japan is a polygenic model of obesity and type 2 diabetes mellitus. KK-A^y^ mice, also named as Yellow KK mice, developed as a result of the dominant mutation of A^y^ gene in KK [33]. As the results, SCE significantly suppressed the increase of both blood glucose and HbA1c levels at doses of 0.25 and/or 0.50% (w/w) in the CE-2 diet without significant changes in body weight and food intake as shown in Table 4.

**Table 4:** Effects of chronic administration of SCE on blood glucose and HbA1c levels in CE-2 diet-fed KK-A^y^ mice.

Group	Dose (%)	Average Food Intake (g/day)			Body Weight (g)		
		0–21 Days			0 Day	15 Days	21 Days
Control	―	7.5 ± 0.2			29.9 ± 0.6	36.6 ± 0.9	39.7 ± 1.1
SCE	0.10	6.9 ± 0.4			29.8 ± 0.5	35.9 ± 1.3	38.4 ± 1.5
	0.25	6.7 ± 0.2			30.0 ± 0.6	37.4 ± 1.0	40.5 ± 1.3
	0.50	6.8 ± 0.1			30.1 ± 0.5	35.5 ± 0.6	39.2 ± 0.8
**Group**	**Dose (%)**	**Blood Glucose (mg/dL)**			**HbA1c (%)**		
		**0 Day**	**15 Days**	**21 Days**	**0 Day**	**15 Days**	**21 Days**
Control	―	206.2 ± 15.7	502.0 ± 33.7	576.3 ± 15.1	3.2 ± 0.1	5.9 ± 0.2	6.9 ± 0.2
SCE	0.10	197.8 ± 17.9	402.3 ± 66.4	436.7 ± 67.6	3.2 ± 0.1	5.0 ± 0.5	5.5 ± 0.6
	0.25	217.7 ± 38.3	335.7 ± 59.5	382.5 ± 64.2	3.2 ± 0.1	4.5 ± 0.2 ^a^	4.8 ± 0.4 ^a^
	0.50	209.3 ± 22.8	217.8 ± 41.4 ^a^	281.7 ± 54.2 ^a^	3.2 ± 0.1	4.1 ± 0.1 ^a^	4.3 ± 0.3 ^a^

Values are means ± SEM (*n* = 6); Significantly different from control, ^a^ *p* < 0.01 (Dunnett’s test); HbA1c: hemoglobin A1c; CE-2: CLEA rodent diet CE-2; KK-A^y^ mice: Kuo Kondo-yellow agouti mice.

The effects of AIN93M purified diet containing 0.03%–0.12% SCE on blood glucose and HbA1c levels of KK-A^y^ mice were evaluated. As shown in Table 5B, administration of the diet containing 0.06% SCE for 11 and 18 days caused significant suppression of both blood glucose and HbA1c levels without significant changes in body weight. Furthermore, 27 days of administration of the diet of 0.12% SCE significantly suppressed not only blood glucose and HbA1c level elevations but body weight gain as well, as shown in Table 5C.

**Table 5:** Effects of chronic administration of SCE on HbA1c levels in AIN93M purified diet-fed KK-A^y^ mice.

Group	(%)	Body Weight (g)			Blood Glucose (mg/dL)			HbA1c (%)		
**(A)**										
		**0 Day**	**11 Days**	**18 Days**	**0 Day**	**11 Days**	**18 Days**	**0 Day**	**11 Days**	**18 Days**
Control	―	27.5 ± 0.3	33.2 ± 0.7	35.4 ± 1.0	243.8 ± 26.8	447.0 ± 44.2	389.8 ± 47.5	3.8 ± 0.1	6.1 ± 0.4	7.2 ± 0.5
SCE	0.03	26.8 ± 0.5	33.2 ± 0.6	35.8 ± 0.9	279.0 ± 46.8	459.0 ± 59.1	415.7 ± 49.7	3.9 ± 0.1	5.6 ± 0.4	6.8 ± 0.5
**(B)**										
		**0 Day**	**11 Days**	**18 Days**	**0 Day**	**11 Days**	**18 Days**	**0 Day**	**11 Days**	**18 Days**
Control	―	27.3 ± 0.4	32.6 ± 0.5	34.1 ± 0.5	231.7 ± 50.3	451.7 ± 33.3	432.5 ± 40.2	3.9 ± 0.1	5.7 ± 0.3	6.7 ± 0.4
SCE	0.06	27.1 ± 0.3	32.3 ± 0.7	34.6 ± 0.6	210.7 ± 29.4	220.5 ± 32.7 ^b^	171.7 ± 6.6 ^b^	4.0 ± 0.1	4.7 ± 0.1 ^a^	5.1 ± 0.2 ^b^
**(C)**										
		**0 Day**	**13 Days**	**27 Days**	**0 Day**	**13 Days**	**27 Days**	**0 Day**	**13 Days**	**27 Days**
Control	―	26.2 ± 0.4	33.1 ± 0.4	38.0 ± 0.5	247.3 ± 22.6	300.1 ± 38.6	305.9 ± 39.8	4.0 ± 0.0	5.1 ± 0.1	5.9 ± 0.2
SCE	0.12	26.9 ± 0.3	32.3 ± 0.3	34.7 ± 0.6 ^b^	299.4 ± 51.3	156.6 ± 9.4 ^b^	184.4 ± 9.5 ^a^	4.0 ± 0.0	4.6 ± 0.1 ^b^	4.7 ± 0.2 ^b^

Values are means ± SEM (*n* = 6–7); Significantly different from control, ^a^ *p* < 0.05, ^b^ *p* < 0.01 (Student’s t-test); Control group was pair-fed the amount of food consumed by SCE-treated group: (**A**) 5.4, (**B**) 4.6, and (**C**) 4.6 g/day, respectively.

**Figure 2:**
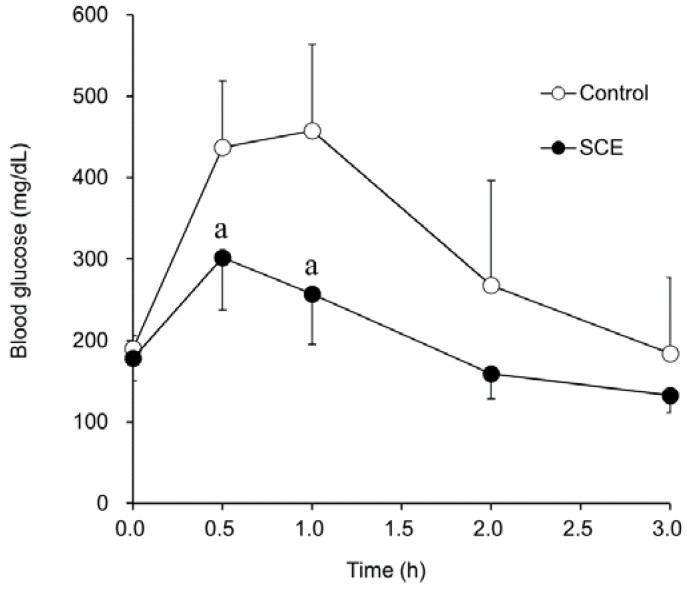
Improvement effect of glucose tolerance after chronic administration of 0.12% SCE in AIN93M purified diet-fed KK-A^y^ mice. Values are means ± SEM (*n* = 7). Significantly different from control, ^a^ *p* < 0.01 (Student’s *t*-test).

**Figure 3:**
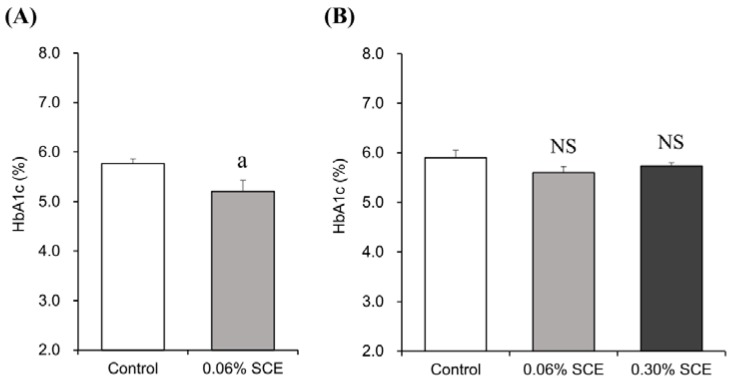
Effects of chronic administration of SCE on HbA1c levels in (**A**) AIN93M purified (**B**) AIN93M/Glc diet-fed KK-A^y^ mice. Values are means ± SEM (*n* = 10); Significantly different from control, ^a^ *p* < 0.05 (Student’s *t*-test); NS: Not significantly different from control (Dunnett’s test); Control group was pair-fed the amount of food consumed by SCE-treated group: (**A**) 5.6 and (**B**) 5.9 g/day, respectively.

Next, the effect of SCE on glucose tolerance was evaluated in glucose-loaded KK-A^y^ mice. Continuous administration to the glucose-loaded KK-A^y^ mice for 27 days of 0.12% SCE in AIN93M purified diet significantly suppressed blood glucose level elevation, apparently improving glucose tolerance (Figure 2). These results suggest that SCE is effective for both inhibiting postprandial glucose elevation and improving glucose tolerance. When the AIN93M/Glc diet, in which all of the digestible glucides were replaced by glucose, containing 0.30% SCE was administered instead of the AIN93M diet, no anti-hyperglycemic activity was observed (Figure 3). Thus, an α-glucosidase inhibitory effect, identified as the mechanism of action in *in vitro* studies was also confirmed in the present *in vivo* examination.

Pint and co-workers reported the stereostructure elucidation and synthesis of **3** [34,35] and also enzymatic inhibitory effects of a series of sulfoniums and their analogs on human recombinant glucosidases, such as catalytic *N*- and *C*-terminal subunits of two retaining exo-glucosidases, maltase-glucoamylase (MGAM) and sucrase-isomaltase (SI), ntMSAM, ctMGAM, ntSI, and ctSI [34,35,36,37]. Activities of the active sulfonium constituents (**1**–**6**) against human intestinal maltase were also evaluated in the present study. As shown in Table 6, **1** (IC_50_ = 4.9 μM), **2** (9.0 μM), **3** (3.9 μM), **4** (3.9 μM), **5** (5.0 μM), and **6** (4.0 μM) inhibited the human maltase as potently as they inhibited rat small intestinal maltase, and their activities were almost equivalent to those of voglibose (1.3 μM) and miglitol (3.7 μM), and even more potent than that of acarbose (15.2 μM).

**Table 6:** IC_50_ values of sulfoniums (**1***–***6**) for human small intestinal maltase.

	IC_50_ (μM)
Salacinol (**1**)	4.9
Neosalacinol (**2**)	9.0
Kotalanol (**3**)	3.9
Neokotalanol (**4**)	3.9
Ponkoranol (**5**)	5.0
Neoponkoranol (**6**)	4.0
Voglibose	1.3
Acarbose	15.2
Miglitol	3.7

Next, the stability of the principal sulfoniums (**1***–***4**) in an artificial gastric juice was examined. More than 96% of each of these sulfoniums survived when they were treated at 37 °C for 1.0 h, as shown in Table 7. More than 90% of them survived even after 3 h of treatment under the conditions, and high stability of these sulfoniums (**1***–***4**) in the artificial gastric juice was observed (Table 7). Their bioavailability through the intestine was also evaluated using an *in situ* rat ligated intestinal loop model. As a result, these sulfoniums were hardly absorbed from the intestine (residual rate (%) of **1**: 97.6 ± 1.8; **2**: 94.5 ± 1.8; **3**: 99.7 ± 2.7; **4**: 96.6 ± 1.7, treated for 2.0 h) as shown in Table 8.

**Table 7:** Stability of sulfoniums (**1***–***4**) in artificial gastric juice.

	Relative Content (% of 0 h)		
	0 h	1.0 h	3.0 h
Salacinol (**1**)	100.0 ± 4.6	100.0 ± 6.9	92.5 ± 6.1
Neosalacinol (**2**)	100.0 ± 6.0	96.5 ± 5.1	93.2 ± 6.2
Kotalanol (**3**)	100.0 ± 4.1	97.3 ± 6.6	91.4 ± 4.6
Neokotalanol (**4**)	100.0 ± 3.3	97.4 ± 3.0	96.5 ± 4.7

Values are means ± SEM (*n* = 3).

**Table 8:** Residual rate of sulfoniums (**1***–***4**) in ligated intestinal loop.

	Relative Content (% of 0 h)		
	0 h	0.5 h	2.0 h
Salacinol (**1**)	100.0 ± 2.4	98.7 ± 2.5	97.6 ± 1.8
Neosalacinol (**2**)	100.0 ± 3.9	101.3 ± 3.0	94.5 ± 1.8
Kotalanol (**3**)	100.0 ± 3.0	98.1 ± 2.6	99.7 ± 2.7
Neokotalanol (**4**)	100.0 ± 2.1	100.0 ± 2.6	96.6 ± 1.7
Miglitol	100.0 ± 1.4	87.6 ± 1.4	52.2 ± 4.7
Glucose	100.0 ± 3.1	32.5 ± 0.5	9.1 ± 0.4

Values are means ± SEM (*n* = 5).

## 4. Conclusions

An antidiabetic effect of hot water extracts of *Salacia chinensis* was observed in *in vivo* studies using KK-A^y^ mice. Elevation of both postprandial blood glucose and HbA1c levels were significantly suppressed by SCE. Glucose tolerance was also improved by the administration of SCE. The active constituents, salacinol (**1**), kotalanol (**3**), and neokotalanol (**4**) inhibited human α-glucosidases as potently as they inhibited rat small intestinal α-glucosidase. The principal sulfonium constituents (**1**–**4**) were highly stable in an artificial gastric juice and were hardly absorbed from the intestine. The results indicate that these sulfoniums are promising leads for a new type of anti-diabetic agents.
